# ArtUnmasked: A Multimodal Classifier for Real, AI, and Imitated Artworks

**DOI:** 10.3390/jimaging12030133

**Published:** 2026-03-16

**Authors:** Akshad Chidrawar, Garima Bajwa

**Affiliations:** Department of Computer Science, Lakehead University, Thunder Bay, ON P7B 5E1, Canada; achidra1@lakeheadu.ca

**Keywords:** AI-generated art detection, artwork authentication, stylistic imitation, Spectral Artifact Identification (SPAI), TagMatch, DINOv3, CLIP, one-shot similarity, Stable Diffusion 2.1, multimodal vision models

## Abstract

Differentiating AI-generated, real, or imitated artworks is becoming a tedious and computationally challenging problem in digital art analysis. AI-generated art has become nearly indistinguishable from human-made works, posing a significant threat to copyrighted content. This content is appearing on online platforms, at exhibitions, and in commercial galleries, thereby escalating the risk of copyright infringement. This sudden increase in generative images raises concerns like authenticity, intellectual property, and the preservation of cultural heritage. Without an automated, comprehensible system to determine whether an artwork has been AI-generated, authentic (real), or imitated, artists are prone to the reduction of their unique works. Institutions also struggle to curate and safeguard authentic pieces. As the variety of generative models continues to grow, it becomes a cultural necessity to build a robust, efficient, and transparent framework for determining whether a piece of art or an artist is involved in potential copyright infringement. To address these challenges, we introduce ArtUnmasked, a practical and interpretable framework capable of (i) efficiently distinguishing AI-generated artworks from real ones using a lightweight Spectral Artifact Identification (SPAI), (ii) a TagMatch-based artist filtering module for stylistic attribution, and (iii) a DINOv3–CLIP similarity module with patch-level correspondence that leverages the one-shot generalization ability of modern vision transformers to determine whether an artwork is authentic or imitated. We also created a custom dataset of ∼24K imitated artworks to complement our evaluation and support future research. The complete implementation is available in our GitHub repository.

## 1. Introduction

Recent advances in generative artificial intelligence have transformed digital synthesis, enabling the creation of realistic and artistically compelling imagery with unprecedented fidelity. Text-to-image diffusion models such as GLIDE, Latent Diffusion, and DALL·E 3 [1,2,3] now translate natural-language prompts into visually coherent artwork, supported by improvements in caption quality [1], controllable generation [4], and scalable transformer architectures [5]. Surveys on diffusion and generative modeling [6,7,8,9] further illustrate their rapid adoption across creative and industrial domains.

However, this generative capability raises profound challenges related to authenticity, copyright, and artistic integrity. Modern diffusion and GAN-based models can reproduce stylistic signatures with such precision that AI-generated artworks often become visually indistinguishable from genuine human-made creations [10,11,12]. This similarity fuels legal and ethical disputes surrounding derivative content, authorship, and ownership of stylistically imitated works. As generative models continue to improve in resolution and photorealism [13,14], existing verification tools struggle to keep pace. Several studies [11,15,16,17,18,19] highlight the increasing difficulty of detecting synthetic imagery—even under spectral, geometric, or forensic scrutiny—underscoring the need for automated, explainable, and general-model detection frameworks.

Building on these challenges, we present ArtUnmasked, a practical framework designed to safeguard artistic authenticity in the era of generative diffusion systems. In our formulation, an artwork belongs to one of three categories: *AI-generated*, *Real*, or *Imitated*. Here, an *Imitated* artwork refers to a real image that replicates or closely reproduces the stylistic or compositional characteristics of another artist’s work, whether produced through an AI-based image-to-image pipeline or through manual recreation.

The framework operates sequentially. First, SPAI functions as a binary filter that separates AI-generated artworks from Real ones using spectral artifact analysis. Only images classified as Real proceed to the second stage. In this stage, a TagMatch-based module identifies the most stylistically aligned reference artists, reducing the candidate search space. Finally, aDINOv3–CLIP similarity module performs semantic and spatial comparison to determine whether the real artwork is Imitated or remains Real.

## 2. Contribution

The main contributions of this work are summarized as follows:

- A lightweight ViT-Tiny SPAI detector that achieves competitive accuracy (94.2%) while reducing computational cost compared to ViT-Base SPAI models, enabling practical large-scale AI-vs-real filtering.
- A scalable tensorized version of TagMatch, enabling fast GPU retrieval across thousands of artists by storing artist–composition signatures in compact sparse matrices rather than string lists.
- A one-shot semantic–spatial comparison module using DINOv3 and CLIP, combining global CLS embedding similarity with patch-level correspondence to distinguish authentic artworks from imitations with fine-grained interpretability.
- A new dataset of ∼24K public-domain imitated artworks, generated using Stable Diffusion 2.1 (img2img) with controlled stylistic perturbations applied to public-domain WikiArt images, supporting future research in imitation detection and evaluation.

## 3. Related Work

### 3.1. Traditional CNN-Based Artwork Analysis

Early work in computational art analysis relied on convolutional architectures for feature extraction and classification. Hua et al. proposed a multiscale pyramidal CNN that fuses global and local cues via Markov random fields to improve artwork representations [20].

Li et al. augmented this direction with a Pyramid Spatial Attention (PSA) module that emphasizes salient regions, which produces better style discrimination [21]. Classical backbones (VGG16, RegNet, and ResNet) remain competitive: Garikipati et al. showed that VGG features plus lightweight classifiers (SVM and random forest) can exceed 90% accuracy for style recognition [22], while Lyberatos et al. demonstrated that RegNet + SVM pipelines achieve robust artist identification even on modest datasets [23]. These results support decoupling feature extraction from classification to enhance flexibility and generalization.

### 3.2. Self-Supervised and Hybrid Graph Methods

To mitigate label scarcity, Luo et al. introduced a dual-teacher contrastive framework that learns strong representations without annotations [24]; Krishnaraja and Prasad documented the risks of overfitting in artist identification with ResNet50 and Inception-v3, underscoring the importance of data quality [25]. At the intersection of graphs and vision, El Vaigh et al. proposed GCNBoost to propagate label information across artist–genre–artwork graphs [26]. Moayeri et al. developed *ArtSavant*, combining deep-match and tag-match routines to flag stylistic imitation by generative models [27]; while promising, such systems remain probabilistic and stop short of deterministic attribution.

### 3.3. Detection of AI-Generated Art

With diffusion and GAN models producing highly realistic visuals, distinguishing AI-generated from genuine artworks has become central. Harsanto et al. and Li–Stamp trained detectors on AIArtBench/WikiArt to capture texture and upsampling artifacts [28,29], and Ring introduced BSP3, a compact CNN for diffusion-trace detection [30]. However, detectors trained on specific generators often degrade on unseen models, motivating the use of domain-general cues and cross-model robustness.

Recent work rethinks cross-generator forgery detection through DINOv3, showing that transformer representations can separate real from AI-generated images by leveraging generator-agnostic authenticity cues that manifest as globally coherent low-frequency structures [31]. Their cross-generator evaluation (e.g., on So-Fake-OOD) further suggests that such cues generalize better than generator-specific artifacts. This supports our design choice of combining spectral filtering (SPAI) with a DINOv3-based semantic–spatial verification stage for imitation analysis.

### 3.4. Watermarking-Based Authenticity Verification

Digital watermarking is widely used for copyright protection and authenticity verification in multimedia security, including robust and reversible watermarking schemes designed to resist geometric attacks [32]. Such methods embed imperceptible signals into visual content to enable later verification of ownership or integrity. Although watermarking techniques provide strong protection when applied at creation time, they are fundamentally dependent on prior embedding and controlled distribution settings.

### 3.5. Transformers, Multimodal, and One-/Zero-Shot Reasoning

Hybrid CNN–ViT models capture both local detail and global structure (Wang–Song) with improved accuracy/F1 [33]; Swin Transformers show strong sensitivity to subtle stylistic differences (Schaerf et al.) [34]; scaling to thousands of artists reveals performance–data coupling (Dobbs et al.) [35]. Multimodal models align images and language: CLIP supports zero- or one-shot transfer by matching visual–textual embeddings without task-specific training [36]. DINOv2 learns robust and transferable descriptors in a self-supervised manner, enabling reliable one-shot similarity under domain shift [37]. These capabilities underpin interpretable, tag-driven filtering and lightweight retrieval in end-to-end attribution pipelines.

### 3.6. Open Gaps

Previous work treats AI detection and artistic attribution as separate problems. Spectral-artifact detectors such as SPAI have not been applied to the AI-versus-real classification of artworks. In contrast, TagMatch-style semantic attribution from ArtSavant [27] is designed for artist and style grouping rather than authenticity assessment. As a result, no existing system performs both (i) AI/real/imitated categorization and (ii) stylistic attribution within a single pipeline. This motivates a unified framework that combines SPAI-based forensic filtering, TagMatch-inspired semantic attribution, and DINOv3-CLIP spatial comparison to distinguish genuine artworks from imitations.

## 4. Dataset

To evaluate the proposed ArtUnmasked framework, we employed three complementary datasets that encompass both authentic and AI-generated artworks as explained in Table 1. Each dataset supports a different stage of the pipeline. The first stage uses SPAI’s frequency-based analysis to detect AI-generated images, the second stage applies TagMatch to identify an artwork’s stylistic origin and potential imitation, and the third stage uses DINO–CLIP feature and patch embeddings to separate authentic artworks from imitated ones by analyzing deeper visual patterns.

### 4.1. WikiArt Dataset (Authentic and Public Domain Artworks)

The WikiArt dataset served as the foundation for both real artwork classification and artist-level stylistic representation. It was obtained using the open-source WikiArt crawler by Lucas David (https://github.com/lucasdavid/wikiart (accessed on 1 August 2025)),which provides metadata for artists, genres, and styles.The complete dataset comprises approximately 200,000 artworks by over 5500 artists spanning a broad range of historical and contemporary movements.

### 4.2. DiffusionDB Dataset (Benchmark for AI vs. Real Classification [SPAI])

To benchmark the spectral detection component, we used the DiffusionDB dataset [38], a large-scale public corpus of text-to-image generation produced using various Diffusion [2] models. From the full DiffusionDB corpus, we sampled 120,000 images to construct the AI-generated class for SPAI training and evaluation. These DiffusionDB samples were used exclusively as the fake (AI-generated) class during SPAI training and paired with authentic artworks from the WikiArt dataset to form a balanced AI-versus-real classification benchmark.

### 4.3. Custom Public-Domain Imitation Dataset

For the real-versus-imitated stage, we curated an imitation dataset generated through image-to-image translation. Using the Stable Diffusion 2.1 model [1] (run locally through the timm interface), a collection of public domain artworks was re-rendered using the img2img pipeline.

Each artwork was modified with controlled stylistic perturbations—such as slight changes in color balance, texture detail, and brushstroke appearance—while preserving the original composition and subject matter. These transformed images are labeled as the *Imitated* class in our framework.

In total, ∼24,000 imitated variants were produced. This dataset serves as the Imitated class for the TagMatch module in the real-versus-imitated evaluation.

**Figure 1:**
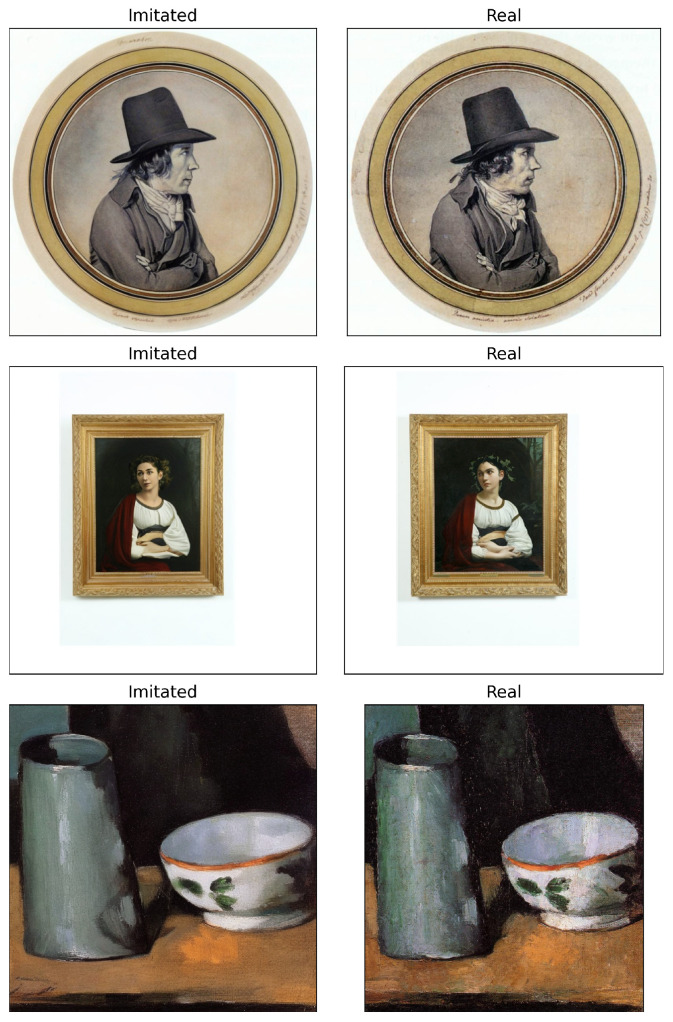
Imitations generated by SD2.1 img2img pipeline vs. the real counterparts.

To illustrate the visual characteristics of these imitated artworks, Figure 1 shows representative examples, with each row showing an imitated artwork generated by img2img (left) alongside its corresponding real artwork (right).

## 5. Materials and Methods

**Figure 2:**
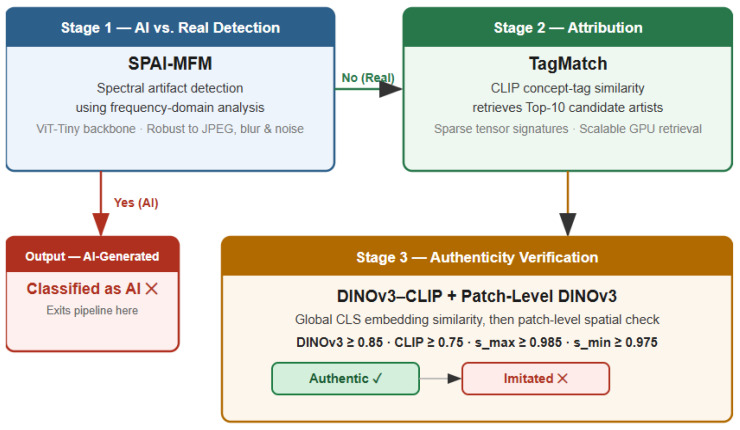
The three-staged ArtUnmasked pipeline.

The proposed ArtUnmasked framework operates as a three-stage verification pipeline, where each stage progressively refines the authenticity decision (Figure 2).

First, a lightweight version of Spectral Artifact Identification (SPAI) [39] distinguishes AI-generated images from genuine artworks using frequency-domain analysis. Images classified as AI-generated are filtered out at this stage, ensuring that only genuine artworks proceed to the next stage of the pipeline.

Second, a TagMatch-based module [27] identifies a small set of reference artists whose stylistic signatures are most similar to the test set. This step reduces the search space to only plausible stylistic matches.

Finally, within this narrowed candidate set, a DINOv3–CLIP module performs global semantic similarity and patch-level spatial comparison. By analyzing both the overall similarity of the embedding and the localized structural consistency, the system determines whether the artwork is authentic or stylistically imitated.

### 5.1. AI vs. Real Classification (SPAI-Based)

The first stage of the proposed pipeline employs a modified version of Spectral Artifact Identification (SPAI) [39] to distinguish AI-generated artwork from genuine human-created images. SPAI operates in the frequency domain by detecting structural inconsistencies introduced during generative image synthesis.

We selected SPAI as the core AI-generated image detector due to its demonstrated state-of-the-art generalization and robustness. As reported by Karageorgiou et al. [39], SPAI achieves an average AUC of 91.0% across 13 diverse generative models, outperforming previous methods by an absolute margin of 5.5%. Unlike artifact-specific detectors that perform well only on particular generators, SPAI maintains consistently high detection accuracy across diffusion-, GAN-, and high-fidelity commercial systems, including SD3, Midjourney, and DALL·E 3. Furthermore, SPAI demonstrates strong robustness to common image perturbations such as JPEG compression, resizing, blur, and noise, which are critical for real-world deployment—a concern broadly shared across vision tasks requiring stable feature representations under corruptions [40]. These properties establish SPAI as a reliable and generator-agnostic foundation for AI-generated artwork detection.

SPAI leverages a pretrained Masked Frequency Modeling (MFM) backbone [41] as its feature extractor. The MFM framework learns frequency-aware representations by randomly masking high- or low-frequency components of input images and training the network to reconstruct the original signal. Through this reconstruction objective, the backbone captures characteristic frequency structures of real images, enabling it to detect spectral deviations introduced by generative processes.

Since the original MFM implementation did not provide a ViT-Tiny variant, we trained a lightweight version following the same training protocol while adapting it to a ViT-Tiny architecture to improve computational efficiency. Compared to the original SPAI implementation based on a ViT-Base backbone, our ViT-Tiny variant substantially reduces model complexity and memory requirements. ViT-Base contains approximately 86M parameters with a 768-dimensional embedding space, whereas ViT-Tiny contains roughly 5–6M parameters with a 192-dimensional embedding. This reduction of more than 15× in the parameter count leads to significantly lower FLOPs and GPU memory consumption, allowing faster training and inference under constrained computational resources. Empirically, processing 20k images required approximately 5 min using our ViT-Tiny SPAI model, compared to 21 min with the ViT-Base backbone under identical hardware conditions.

The fine-tuned MFM backbone serves as a feature extractor for our lightweight ViT-Tiny SPAI classifier. The detector is trained on a balanced dataset consisting of 100k diffusion-generated images from DiffusionDB and 100k genuine artworks from WikiArt. During inference, SPAI functions as a binary filtering stage: images classified as AI-generated are excluded from further analysis, while only those identified as real proceed to the subsequent stages of stylistic attribution and imitation verification.

Unlike the original SPAI implementation, which employs a large-scale pretrained ViT-Base backbone before detector training, our lightweight variant uses a ViT-Tiny backbone, pretrained under more constrained compute and data settings. Consequently, while our implementation does not aim to fully replicate SPAI’s broad cross-generator evaluation, it demonstrates strong performance within our experimental domain, achieving 93.2% test accuracy on DiffusionDB. These findings indicate that the SPAI spectral learning principle remains effective even under reduced model capacity, while larger-scale pretraining and a wider multi-generator evaluation remain promising directions for further improving cross-model robustness.

### 5.2. Artist Tag Signature Generation (TagMatch-Based)

If the test set contains multiple images, the tag match serves as an artist-style classifier; otherwise, the image proceeds directly to the One-Shot Semantic and Spatial Comparison (DINOv3–CLIP) stage. Our artist-signature construction follows the TagMatch framework proposed by Moayeri et al. [27], which emphasizes interpretable attribution through structured visual concepts. We adapt this methodology to seamlessly integrate into our ArtUnmasked pipeline and extend it with a tensorized storage format to enable large-scale fast retrieval.

#### 5.2.1. Vocabulary Construction

We define a structured vocabulary of aspect-concept pairs (e.g., *Line: Geometric*, *Color: Monochromatic*, *Texture: Rough*). These pairs represent the perceptual, stylistic, and compositional attributes of artworks. For every aspect—concept entry—we generated multiple descriptive caption templates using ChatGPT [42] and Vicuna-13B [43]. Each template expresses how the concept may manifest visually, such as:

- “A composition built from geometric forms”;
- “An artwork defined by geometric shapes”;
- “A design emphasizing geometric precision”.

To preserve linguistic consistency, all templates follow one of five controlled syntactic structures “A composition…,” “An artwork…,” “A design…,” “A painting…,” “An image…” combined with predicate patterns such as “built from,” “defined by,” “shaped by,” “marked by,” and “inspired by”.

We embed each descriptor (aspect–concept) using the CLIP text encoder and *average* embeddings across all templates belonging to the same concept, yielding a stable, semantically consistent embedding for every aspect–concept pair.

#### 5.2.2. Atomic Tag Extraction

For each artwork image, we compute cosine similarities between its CLIP image embedding and all descriptor embeddings. Scores are normalized within each aspect using the *z*-score, and descriptors z≥1.5 are retained as atomic tags.

For each artist *a*, the atomic tags that appear in at least three artworks are preserved as the common atomic tags. For each image, if the number of tags exceeds 25, the tags are sorted in descending order based on their *z*-scores, and only the top 25 are retained as the atomic tags of that image.

**Figure 3:**
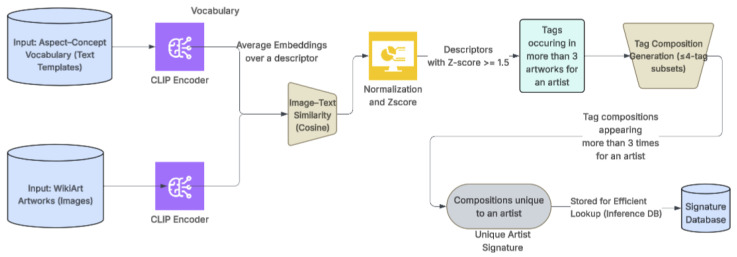
Overview of the tag-based artist signature generation pipeline.

These tags capture stylistic consistency across the artist’s set of work and form the foundation for the construction of unique signatures (see Figure 3).

#### 5.2.3. Tag Compositions and Efficient Signature Representation

After extracting meaningful tags for each artwork, we identify small groups of tags (pairs or triplets) that frequently co-occur within the same image. For each artist, we scan all artworks and record tag compositions that appear in at least three images. These recurring compositions form the artist’s stylistic signature, capturing consistent patterns throughout their body of work, following the TagMatch framework [27].

To enable scalable retrieval across thousands of artists, we store these signatures in a compact numerical format rather than raw text. Each unique tag composition is assigned a global integer identifier, and artist–composition frequencies are stored as sparse matrices. We also maintain a rarity vector that indicates how many artists use each composition. Formally, the representation consists of

{composition_vocab,artist_to_id,rarity_vector,artist_composition_matrix}.

This structured sparse representation reduces memory overhead and enables efficient computation of GPU-based similarity while preserving interpretability.

### 5.3. TagMatch-Based Artist Filtering

After constructing signature sets for every artist, the next step is to determine which reference artists are most stylistically similar to a given test set of images. The procedure follows the method of TagMatch [27] but is rewritten here in a simplified and more interpretable form.

Step 1: Build a signature for the test set:

For a test set *T* of images, we extract atomic tags and tag combinations using the same procedure used for reference artists. This produces the following:

- A test signature $\Sigma_{T}$; and
- A frequency count for each tag or composition.

Step 2: Find matched tags with each artist:

For every reference artist *a*, we compute the intersection,

$$M_{a}=\Sigma_{T}\cap \Sigma_{a},$$

which lists the tags or compositions that appear in both the test set and the artist’s signature.

For each matched tag, we compute its *rarity*, defined as the number of artists who also use that tag. Rare tags are more discriminative, so we sort $M_{a}$ so that rare tags are considered first.

Step 3: Measure stylistic similarity using frequency deviation: For every matched tag, we compare the following:

- How frequently the test set uses that tag; and
- How frequently the reference artist uses that tag.

Let $f_{T}\left(s\right)$ denote the fraction of test images containing the tag *s*, and $f_{a}\left(s\right)$ denote the fraction of artists *a* containing the same tag. The difference

$$\Delta_{a}\left(s\right)=\left|f_{T}\left(s\right)-f_{a}\left(s\right)\right|$$

quantifies how similarly the tag is used. A lower value of $\Delta_{a}\left(s\right)$ indicates a closer stylistic alignment between the test set and the artist *a*.

Step 4: Score each artist and keep the best matches:

Each matched tag receives a score that combines its rarity and its stylistic deviation as follows:

$$\mathrm{Score}\left(s,a\right)=\lambda \;U\left(s\right)+\left(1-\lambda \right)\;\Delta_{a}\left(s\right),$$

where U(s) is the rarity of the tag *s*, and λ∈[0,1] balances the two terms.

For each artist, we retain only the *k* best-matched tags (default k=10). Artists with fewer *k* matches are discarded because they lack sufficient stylistic overlap to be reliable candidates.

The remaining artists are ranked by the average score of their top *k* tags. The 10 artists with the lowest scores form the final set of candidates. Each test artwork is then compared against the artworks of these shortlisted artists using DINOv3-CLIP similarity to detect potential imitations.

### 5.4. One-Shot Semantic and Spatial Comparison (DINOv3–CLIP)

**Figure 4:**
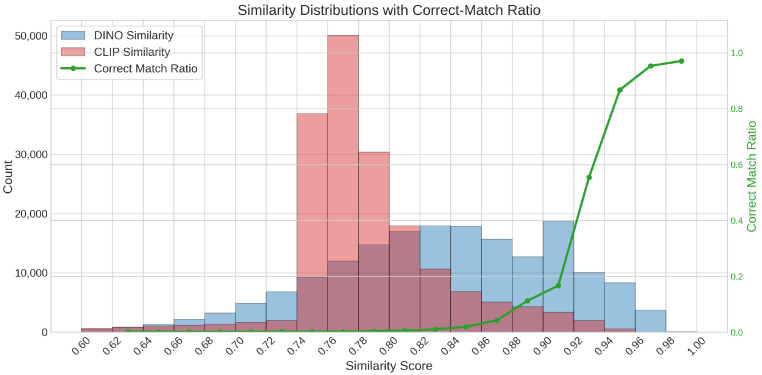
DINOv3 and CLIP cosine similarity analysis for 24,000 imitation-real artwork pairs. For each similarity threshold, the green curve reports the fraction of pairs that match their authentic counterpart.

After obtaining the top-10 candidate artists from TagMatch, we apply a finer verification step using DINOv3 [31] and CLIP [36] embeddings. The aim is to check whether the test artwork is spatially and semantically similar to any artwork from the shortlisted artists. For each test image, we compute CLS embeddings and compare them with embeddings of real artworks using cosine similarity. As shown in Figure 4, we compute DINOv3 and CLIP cosine similarities for each of the 24k imitation-real pairs.

Pairs with DINOv3 similarity ≥0.85 and CLIP similarity ≥0.75 are treated as strong candidates and retained for further consideration. Among all retained pairs, we select the image pair that achieves the highest combined cosine similarity (based on DINOv3 and CLIP scores). This selected pair is then forwarded to the patch-level DINOv3 comparison for detailed spatial verification.

Pairs that do not satisfy either threshold are labeled as non-imitated and are not processed further.

### 5.5. Patch-Level Spatial Comparison

Patch-level correspondence is essential because global embedding similarity alone cannot reliably distinguish authentic artworks from stylistic imitations. Two images may share high semantic similarity at the CLS level—reflecting similar subject matter, composition, or color palette—while still differing in localized structural details. Authentic pairs (e.g., exact copies or minimally altered versions) tend to preserve consistent spatial alignment across most image regions. In contrast, imitated artworks often introduce subtle but spatially inconsistent variations in texture, brushstroke patterns, or fine structural details, even when overall similarity remains high.

Recent analysis of DINOv3-based forgery detection shows that authenticity cues are not restricted to global CLS tokens but emerge as distributed patterns in spatial patch tokens [44]. In particular, cross-generator studies demonstrate that generator-agnostic signals are encoded within localized structural representations. This observation motivates our use of patch-level DINOv3 similarity to verify spatial consistency between the candidate artwork pairs.

To capture these differences, we extract patch embeddings from the DINOv3 feature maps and compute the cosine similarity across all spatial tokens. From these values, we derive a maximum similarity score $s_{\max}$ and a minimum similarity score $s_{\min}$. While $s_{\max}$ reflects the strongest regional alignment, $s_{\min}$ is more sensitive to localized deviations. Authentic pairs typically exhibit uniformly high similarity across patches, whereas imitations often contain specific regions where similarity drops, making $s_{\min}$ particularly informative for detecting subtle structural inconsistencies.

**Figure 5:**
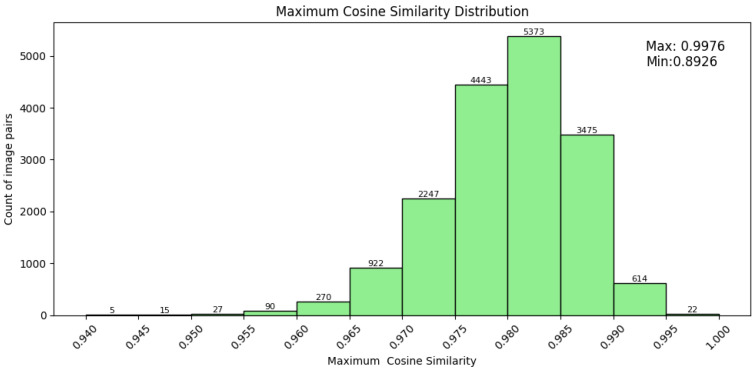

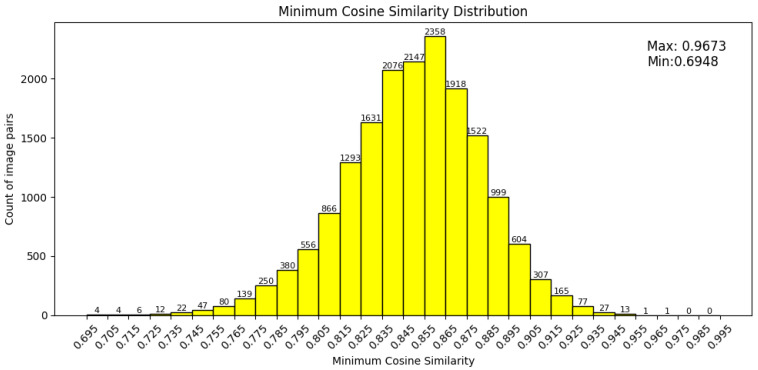
Distributions of the maximum ($s_{\max}$) and minimum ($s_{\min}$) DINOv3 patch-similarity scores computed over 24k imitation–real pairs.

Based on the observed distributions (Figure 5), we classify a pair as authentic if

$$s_{\max}\geq 0.985\;\mathrm{and}\;s_{\min}\geq 0.975.$$

Otherwise, the pair is labeled as imitated.

**Figure 6:**
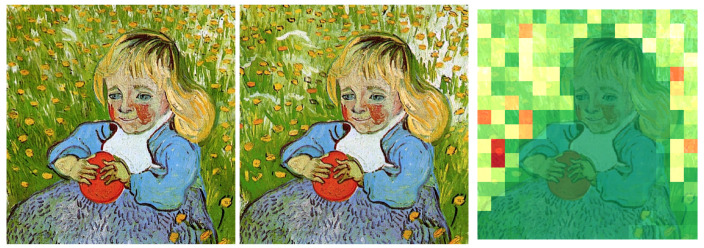
Patch-level DINOv3 similarity analysis. (**left**) Imitated artwork. (**middle**) Original artwork. (**right**) Patch-wise cosine similarity heatmap (green: high similarity; red: low similarity). The global similarities for this example are DINOv3 = 0.98 and CLIP = 0.93.

Figure 6 illustrates this process, with normalized patch-wise DINOv3 similarities visualized as a heatmap to highlight differences between real and imitated images.

This patch-level verification step, therefore, provides a spatial verification score along with a patch-wise explanation, making the imitation decision transparent and interpretable.

## 6. Results and Evaluation

To evaluate the effectiveness of the proposed ArtUnmasked framework, we conduct experiments on two complementary tasks; distinguishing AI-generated images from real artworks and detecting stylistic or compositional imitation across artists. Table 2 summarizes the quantitative evaluation metrics across all stages of the proposed framework.

### 6.1. AI-Generated Image Detection

We evaluated the lightweight SPAI module on two datasets: DiffusionDB and AIArtBench.

In DiffusionDB, which contains diffusion-generated images paired with real artworks, ViT-Tiny-based SPAI achieves an overall test accuracy of 93.2%.

We further evaluate generalization using the AIArtBench dataset [45], which includes a balanced mix of diffusion- and GAN-based synthetic artworks alongside genuine human-created pieces. In this benchmark, the model achieves an average test accuracy of 75%, demonstrating reasonable robustness between datasets despite domain differences.

### 6.2. Robustness of SPAI Under Image Perturbations

To evaluate the robustness of the SPAI module under realistic degradations, we conducted additional experiments on a balanced subset of 1000 real WikiArt images and 1000 AI-generated DiffusionDB images. We assessed performance under JPEG compression (quality 85 and 70) and Gaussian noise (σ=5).

As shown in Table 3, SPAI demonstrates strong resilience to JPEG compression, with accuracy remaining above 94% and AUC above 0.987 at both quality levels. When Gaussian noise is introduced, performance decreases moderately (2.15% drop in accuracy and 0.0062 drop in AUC), but remains high overall, indicating that SPAI retains discriminative spectral cues under realistic perturbations.

### 6.3. Imitation and DINO–CLIP Authenticity Verification

We evaluate imitation detection using the custom public-domain imitation dataset described in Section 4.3. First, the TagMatch module identifies stylistically similar reference artists. In this dataset, TagMatch achieves a Top-10 accuracy of 98.6% (Table 4), indicating that co-occurring tag compositions effectively capture consistent stylistic patterns across artists. The shortlisted candidates are then refined using DINOv3–CLIP semantic similarity. Restricting comparisons to the top-10 TagMatch predictions, this stage achieves 96.2% accuracy with thresholds of 0.85 (DINOv3) and 0.75 (CLIP).

**Figure 7:**
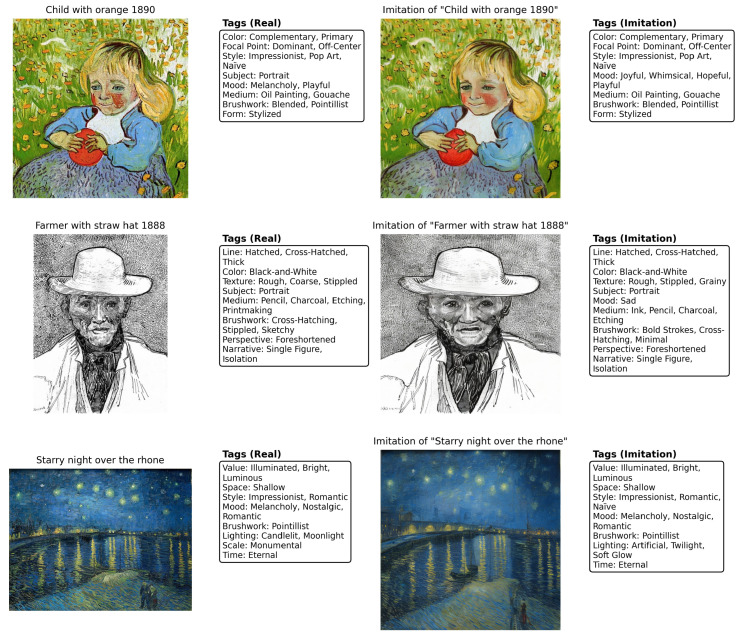
Real–imitation artwork pairs with TagMatch atomic tags. The left image shows the authentic artwork, and the right shows its imitation. Extracted tags highlight preserved stylistic attributes with minor local variations.

Finally, patch-level DINOv3 correspondence is applied to high-similarity pairs. Using spatial similarity bounds of 0.985 (maximum) and 0.975 (minimum), this verification step achieves 100% correctness on the thresholded pairs. Together, these results demonstrate that the coarse-to-fine TagMatch with DINOv3–CLIP pipeline reliably detects stylistic imitation while maintaining high precision. Figure 7 shows representative real–imitation pairs with their extracted atomic tags.

## 7. Ablation Study

### 7.1. Broad Evaluation on the Eval Dataset

Table 5 shows the per-class performance of the full ArtUnmasked pipeline on a held-out evaluation set of 8 500 images. TagMatch correctly retrieves the source artist within the top-10 candidates for 98.6% of imitations and 100% of real WikiArt artworks. DINOv3–CLIP attribution succeeds for 98.1% of imitations, while the false-positive rate on pure AI-generated images remains at 1.3%, confirming that the pipeline rarely attributes diffusion output to a real artist.

### 7.2. CLIP Backbone Ablation

To assess the impact of the CLIP backbone on TagMatch accuracy and processing time, we sweep three open-CLIP variants with fixed hyperparameters (top-25 tags per image during training, top-15 at test time, compositions up to size 3). Table 4 summarizes the results. All three backbones achieve Top-10 accuracy ≥ 98.6%, confirming that TagMatch performance is robust to backbone choice. ViT-L-14 achieves the highest Top-5 accuracy (95.9%) while ViT-B-16 offers the fastest total runtime (4.98 min vs. 7.13 min for ViT-L-14 and 12.32 min for ViT-H-14).

### 7.3. Comprehensive Comparison with Related Work

To position our unified framework within the broader literature, we provide a structured comparison across three related but distinct tasks: (i) AI-generated artwork detection, (ii) artist attribution/painter recognition, and (iii) style classification. Since prior work evaluated different tasks across varying experimental setups, we separated the comparisons accordingly to ensure clarity and fairness.

Table 6 shows a comparison of our work with other AI-generated image detection frameworks in the literature. Our SPAI-based module operates in the spectral domain and is integrated within a hierarchical filtering pipeline, enabling robust detection across generated artwork variants.

The original TagMatch framework reports Top-5 and Top-10 retrieval accuracies of 82.5% and 88.4%, respectively, on a held-out split of the WikiArt dataset. In contrast, we evaluate our adapted TagMatch module under a large-scale imitation retrieval setting consisting of 24K real WikiArt artworks forming the gallery and 24K generated imitations used as queries. For each imitation, retrieval is performed against the full 24K real gallery, and performance is measured based on whether the ground-truth artist appears within the Top-*K* retrieved results.

As shown in Table 7, our adapted TagMatch module achieves 95.9% Top-5 and 98.6% Top-10 accuracy under the imitation retrieval setting.

Table 8 presents representative style classification accuracies reported on WikiArt dataset, operating under closed-set style recognition settings and are included to contextualize the performance range of artwork recognition systems in the literature.

## 8. Conclusions

In this work, we presented ArtUnmasked, a unified multi-modal framework for determining whether an artwork is real, AI-generated, or stylistically imitated. The system integrates spectral-domain artifact detection via a lightweight SPAI–ViT-Tiny model, a scalable TagMatch-based artist attribution module, and a semantic–spatial comparison stage using DINOv3-CLIP embeddings.

Our results demonstrate that a combination of frequency cues, concept-level stylistic signatures, and fine-grained spatial correspondence provides a reliable basis for artwork authentication. The proposed tensorized TagMatch formulation enables efficient large-scale retrieval across thousands of artists, while the hybrid spectral–semantic–spatial pipeline remains robust to stylistic variation and generative-model diversity.

ArtUnmasked serves as a practical foundation for future systems that preserve artistic authenticity, detect copyright infringement, and support art institutions in validating digitized cultural content with minimal computational overhead.

## 9. Limitations and Future Work

Although ArtUnmasked demonstrates strong performance across AI detection and imitation verification tasks, some limitations remain. First, the lightweight SPAI classifier relies on an MFM-pretrained ViT backbone whose robustness is constrained by the scale and diversity of the real-world data used during pretraining. Although current MFM training leverages a large corpus of artworks, extending this pretraining to broader, more heterogeneous real-image datasets could further improve spectral generalization and robustness.

Second, the TagMatch module is highly dependent on the quality and coverage of the CLIP-based visual concept vocabulary. Uncommon, abstract, or highly nuanced artistic motifs may be underrepresented, reducing the discriminative power for certain artists. A richer and more distinctive vocabulary—potentially constructed from manually curated tags or expanded LLM-generated descriptors—could further enhance interpretability and stylistic coverage. Additionally, fine-tuning a CLIP encoder specifically on artistic imagery can improve both vocabulary generation and tag-matching accuracy.

Third, although robust, DINO-based similarity metrics can be sensitive to composition changes, cropping, or heavy abstraction. This sensitivity may affect imitation detection in highly abstract artworks, where spatial correspondence does not cleanly align with semantic similarity.

## Figures and Tables

**Table 1:** Summary of datasets and their role in the ArtUnmasked framework.

Dataset	Type	Source/Model	Image Count	Usage in Framework
WikiArt (Non–Public-Domain)	Real	Lucas David crawler [27]	180,000	SPAI training/testing (real class)
WikiArt (Public Domain)	Real	Lucas David crawler [27]	∼24,000	TagMatch reference base; source images for imitation generation
Diffusion DB [38]	AI-Generated	Diffusion Models [2]	120,000 total	SPAI training/testing (AI-generated)
SD2.1 (Img2Img)	Imitations	Stable Diffusion [2]	∼24,000	TagMatch imitation dataset; DINOv3–CLIP real-vs-imitation classification

**Table 2:** Quantitative evaluation metrics of the ArtUnmasked framework.

Module	Metric	Value	Dataset
SPAI (ViT-Tiny)	Accuracy	93.2%	DiffusionDB
SPAI (ViT-Tiny)	AUC	99.1%	DiffusionDB
SPAI (ViT-Tiny)	Average Precision	98.2%	DiffusionDB
SPAI (ViT-Tiny)	Accuracy	75%	AI-ArtBench
TagMatch	Top-10 Accuracy	98.6%	Imitation Dataset
DINOv3–CLIP	Retrieval Accuracy	96.2%	Imitation Dataset
Patch-Level Verification	Classification Accuracy	100%	Thresholded Pairs

**Table 3:** Robustness evaluation of SPAI under image perturbations (1000 Real + 1000 AI images).

Setting	Accuracy	F1 (Avg)	AUC	AP
Original Images	95.50%	95.50%	0.9905	0.9920
JPEG (Q = 85)	94.25%	94.25%	0.9879	0.9894
JPEG (Q = 70)	94.25%	94.25%	0.9879	0.9894
JPEG (Q = 85) + Gaussian Noise (σ=5)	93.35%	93.35%	0.9843	0.9863

**Table 4:** CLIP backbone ablation on ArtUnmasked. Signatures built with top-25 tags/image and combinations up to size 3; test inference uses top-15 tags/image. The timing columns show wall-clock seconds per step.

Model	Top-5	Top-10	Load (s)	Embed (s)	Cosine (s)	Sig (s)	TagMatch (s)	Total (min)
ViT-H-14	89.04%	98.63%	7.3	312.1	29.9	32.4	19.7	12.32
ViT-B-16	90.41%	98.63%	1.5	125.4	14.2	26.2	16.8	4.98
ViT-L-14	95.89%	98.63%	4.3	163.8	22.0	28.1	18.1	7.13

**Table 5:** Per-class evaluation of TagMatch and DINOv3–CLIP attribution on the broad eval dataset (8500 images: 5000 imitations, 2500 real (WikiArt overlap), 1000 DiffusionDB).

Class	N	Top-5 TagMatch	Top-10 TagMatch	DINO + CLIP Attr. Rate
Imitation	5000	94.4%	98.6%	98.1%
Real (WikiArt)	2500	100.0%	100.0%	100.0%
AI-Generated	1000	N/A	N/A	1.3% ^†^

^†^ False-positive rate: fraction of diffusion images matched to any reference artist.

**Table 6:** Comparison of our work with other AI-generated image detection frameworks across different evaluation settings.

Method	Dataset	Setting	Accuracy (%)
**SPAI (Ours)**	WikiArt + DiffusionDB	Artwork detection	**93.2**
Detecting AI-Generated Artworks With DL [28]	45K real/45K AI	Binary classification	98.23 (Avg)
Detecting AI-generated Artwork [29]	WikiArt + AI-ArtBench	Binary/Multi-class	97.58/82.08
DINOv3 Cross-Generator [44]	GenImage	Cross-generator OOD	92.6

**Table 7:** Artist attribution comparison on WikiArt dataset.

Method	Top-5 (%)	Top-10 (%)
TagMatch (Original, held-out WikiArt) [27]	82.5	88.4
Synergy (RegNetY-1.6MF + SVM) [23]	85	–
**TagMatch (Ours, 24K imitation → 24K gallery)**	**95.9**	**98.6**

**Table 8:** Reported WikiArt style classification performance from literature. Results are taken from original publications and may reflect different experimental protocols.

Method	Accuracy (%)
Self-Supervised Dual-Teacher KD [24]	63.37
Attention-CNN (CNN + PSA) [21]	91.52
**TagMatch (Ours, artist attribution)**	**98.6** (top-10)

## Data Availability

The datasets used in this study are publicly available. The WikiArt dataset was collected using the Lucas-David WikiArt crawler (https://github.com/lucasdavid/wikiart, accessed on 7 March 2026). The AI-ArtBench dataset was obtained from Kaggle (https://www.kaggle.com/datasets/ravidussilva/real-ai-art, accessed on 7 March 2026). The DiffusionDB dataset was obtained from its official repository (https://poloclub.github.io/diffusiondb/, accessed on 7 March 2026). The Stable Diffusion 2.1 generated dataset created by the authors will be available via the project repository at https://github.com/AkshadC/ArtUnmasked, accessed on 7 March 2026.
